# Arachidonic acid drives adaptive responses to chemotherapy-induced stress in malignant mesothelioma

**DOI:** 10.1186/s13046-021-02118-y

**Published:** 2021-11-02

**Authors:** Mario Cioce, Claudia Canino, Harvey Pass, Giovanni Blandino, Sabrina Strano, Vito Michele Fazio

**Affiliations:** 1 Department of Medicine, R.U. in Molecular Medicine and Biotechnology, University Campus Bio-Medico of Rome, 00128 Rome, Italy; 2 Division of General Thoracic Surgery, Department of Cardiothoracic Surgery, NYU Langone Medical Center, New York, NY USA; 3 Present Address: Radiation Oncology Unit, UPMC Hillmann Cancer Center, San Pietro Hospital FBF, Rome, Italy; 4 Oncogenomic and Epigenetic Unit, IRCCS Regina Elena National Cancer Institute, 00144 Rome, Italy; 5 SAFU Unit, Department of Research, Diagnosis and Innovative Technologies, IRCCS Regina Elena National Cancer Institute, 00144 Rome, Italy; 6 Institute of Translational Pharmacology, National Research Council of Italy (CNR), 00133 Rome, Italy; 7 Laboratory of Oncology, Fondazione IRCCS Casa Sollievo della Sofferenza, 71013 San Giovanni Rotondo, Italy

**Keywords:** Arachidonic acid, cPLA2, NFkB, Chemoresistance, ALDH, Spheroids, Malignant pleural mesothelioma, MPM

## Abstract

**Abstract:**

Background

High resistance to therapy and poor prognosis characterizes malignant pleural mesothelioma (MPM). In fact, the current lines of treatment, based on platinum and pemetrexed, have limited impact on the survival of MPM patients. Adaptive response to therapy-induced stress involves complex rearrangements of the MPM secretome, mediated by the acquisition of a senescence-associated-secretory-phenotype (SASP). This fuels the emergence of chemoresistant cell subpopulations, with specific gene expression traits and protumorigenic features. The SASP-driven rearrangement of MPM secretome takes days to weeks to occur. Thus, we have searched for early mediators of such adaptive process and focused on metabolites differentially released in mesothelioma vs mesothelial cell culture media, after treatment with pemetrexed.

**Methods:**

Mass spectrometry-based (LC/MS and GC/MS) identification of extracellular metabolites and unbiased statistical analysis were performed on the spent media of mesothelial and mesothelioma cell lines, at steady state and after a pulse with pharmacologically relevant doses of the drug. ELISA based evaluation of arachidonic acid (AA) levels and enzyme inhibition assays were used to explore the role of cPLA2 in AA release and that of LOX/COX-mediated processing of AA. QRT-PCR, flow cytometry analysis of ALDH expressing cells and 3D spheroid growth assays were employed to assess the role of AA at mediating chemoresistance features of MPM. ELISA based detection of p65 and IkBalpha were used to interrogate the NFkB pathway activation in AA-treated cells**.**

**Results:**

We first validated what is known or expected from the mechanism of action of the antifolate. Further, we found increased levels of PUFAs and, more specifically, arachidonic acid (AA), in the transformed cell lines treated with pemetrexed. We showed that pharmacologically relevant doses of AA tightly recapitulated the rearrangement of cell subpopulations and the gene expression changes happening in pemetrexed -treated cultures and related to chemoresistance. Further, we showed that release of AA following pemetrexed treatment was due to cPLA2 and that AA signaling impinged on NFkB activation and largely affected anchorage-independent, 3D growth and the resistance of the MPM 3D cultures to the drug.

**Conclusions:**

AA is an early mediator of the adaptive response to pem in chemoresistant MPM and, possibly, other malignancies.

**Supplementary Information:**

The online version contains supplementary material available at 10.1186/s13046-021-02118-y.

## Background

Malignant Pleural Mesothelioma (MPM) is a cancer of the mesothelial lining that protects multiple internal organs such as the lungs and heart [1]. Diagnosis is often late because of its silent clinical course. Despite some encouraging results with immune checkpoint inhibitors [2, 3], a combination of cisplatin and pemetrexed (pem), is still the most diffuse therapeutic approach. This latter holds limited success and contributes to the poor prognosis of MPM patients [4, 5]. Pemetrexed is an anti-folate targeting thymidylate synthase, dihydrofolate reductase, and glycinamide ribonucleotide formyltransferase and thus inhibiting the formation of purine and pyrimidine nucleotides [6]. Pem may also exert metabolic reprogramming, through activation of the AMPK alpha subunit and modulation of glucose hemostasis [7, 8]. A main challenge in the therapeutic management of MPM is the very pronounced resistance to therapy, an intrinsic feature of the tumor cells [9]. Resistance of mesothelioma cells to chemotherapy is a result of complex phenomena. Others and we have shown that emergence of chemoresistant cell subpopulations within the treated MPM samples is supported by the acquisition of a senescence associated secretome rearrangement, subsequent to the onset of a Senescence-Associated-Secretory-Phenotype (SASP) [10–14]. This latter was characterized by the release, in the conditioned media, of a number of cytokines and growth factors, which promoted the emergence of the chemo-resistant cell subpopulations expressing high levels of the detoxifying enzyme Aldehyde Dehydrogenase (ALDH) [13, 15]. Activation of the NFkB and STAT3 pathways was relevant to the onset of such a response [13, 16]. Since the SASP-driven secretome rearrangement takes days to occur, searching for earlier mediators of such a response may be relevant and may hold translational potential. Lipid metabolites are receiving tremendous attention as emerging key regulators of cancer cell behavior [17, 18]. Increase of unsaturated lipids holds protumorigenic properties. For example, there is evidence that cisplatin-induced polyunsaturated fatty acids (PUFAs) mediate chemoresistance both in co-culture and in animal models [19]. A high level of lipid desaturation was identified as a trait of ovarian Cancer Stem Cells [20]. Arachidonic acid (AA), a representative ω-6 polyunsaturated fatty acid (PUFA), is one of the most abundant fatty acids released into a wound site during skin injury [21]. It plays a role in the regeneration of various types of tissues, including gut, after ischemia [22]. According to these early evidence, AA could stimulate survival and proliferation of hematopoietic stem cells, mesenchymal stem cells, and embryonic stem cells [23]. More recently, AA promoted intestinal regeneration in irradiated mice by positively modulating survival of stem –like epithelial cell subpopulations and by negatively modulating their differentiation [24]. We have hypothesized that the metabolic perturbation following pemetrexed treatment of mesothelioma cells may impinge on the onset of the SASP-driven secretome rearrangement. Metabolic changes may happen very early after drug treatment and may produce signaling molecules. Additionally, metabolites have the potential to function as fast acting signaling molecules, in a paracrine way [25, 26]. Thus, we have investigated the extracellular metabolic profile of pem-treated mesothelioma cells, with the aim of identifying metabolites released by chemotherapy-treated cells and not by untransformed, chemo-sensitive ones. We found that cPLA2-dependently, early released AA, mediated the adaptive response of MPM cells to pemetrexed. AA treatment fully recapitulated the cell subpopulation rearrangements and the gene expression changes instigated by pemetrexed driving acquisition of protumorigenic traits. This was mediated by NFkB activation and initiated the SASP-related secretome changes.

## Methods

### Cell lines and treatments

The human MPM cell lines MSTO211H, ZL34, and SPC-212 were from the Sigma-Aldrich (St. Louis, MO, USA). MPP-89 were from the Istituto Nazionale per la Ricerca sul Cancro, Genova, Italy. LP9, HP1, NCI-H2373, NCI-H2052, NCI-H2452, NCI-H28, NCI-H2595, NCI-H2596, were from Prof. Harvey Pass, NYU, New York, USA. Human mesothelial cells (HMC) (from four pooled healthy donors) were from Zen-Bio, Inc. Durham, NC, USA). The cell lines were cultured as monolayers at 37°C and 5% CO2 in DMEM/F12+GLUTAMAX supplemented with 10% non-heat-inactivated FBS (fetal bovine serum; Invitrogen-Gibco, Carlsbad, CA, USA). All the cell lines were in house-tested for mycoplasma contamination by using commercially available PCR-based assay (R&D Systems). Arachidonic acid (AA) was from Cayman Chemical (Ann Arbor, Michigan, USA). Pemetrexed, A-64077 (Zileuton) and indomethacin (NSC-77541) were from SelleckChem (Houston, TX, USA). Arachidonyl-trifluoromethyl ketone (AACOCF3) was from Santa Cruz Biotechnology (Dallas, Texas, USA.) and the ω3-PUFA derivative AVX-001 was from InvivoChem (Libertyville IL, USA). All the compounds were dissolved according to the manufacturer’s instructions. Ctrl was saline solution (pem, indomethacin, A-64077). AA was firstly diluted in DMSO, then in saline, accordingly. Before adding AA, with or without the cPLA2 inhibitors, cells were shortly (90 min) starved in DMEM-F12 + Glutamax supplemented with 1% bovine serum albumin (BSA).

### Metabolomic analysis

#### Sample preparation

The sample preparation process was carried out using an automated MicroLab STAR® system (Hamilton). Recovery standards were added prior to the first step in the extraction process for QC purposes. Sample preparation was conducted using sequential organic and aqueous extractions to remove the protein fraction while allowing maximum recovery of small molecules. Samples were processed through both Liquid chromatography/Mass Spectrometry (LC/MS) and Gas chromatography/Mass Spectrometry (GC/MS).

#### Liquid chromatography/mass spectrometry (LC/MS, LC/MS^2^)

The LC/MS portion of the platform was based on a Waters ACQUITY UPLC and a Thermo-Finnigan LTQ mass spectrometer, which consisted of an electrospray ionization (ESI) source and linear ion-trap (LIT) mass analyzer. The sample extract was split into two aliquots, dried, then reconstituted in acidic or basic LC-compatible solvents, each of which contained 11 or more injection standards at fixed concentrations. One aliquot was analyzed using acidic positive ion optimized conditions and the other using basic negative ion optimized conditions in two independent injections using separate dedicated columns. Extracts reconstituted in acidic conditions were gradient eluted using water and methanol both containing 0.1% Formic acid, while the basic extracts, which also used water/methanol, contained 6.5 mM Ammonium Bicarbonate. The MS analysis alternated between MS and data-dependent MS^2^ scans using dynamic exclusion.

#### Gas chromatography/mass spectrometry (GC/MS)

The samples destined for GC/MS analysis were dried under vacuum for a minimum of 24 h prior to being derivatized under dried nitrogen using bistrimethyl-silyl-triflouroacetamide (BSTFA). The GC column was 5% phenyl and the temperature ramp is from 40° to 300 °C in a 16 min period. Samples were analyzed on a Thermo-Finnigan Trace DSQ fast-scanning single-quadrupole mass spectrometer using electron impact ionization.

#### Accurate mass determination and MS/MS fragmentation (LC/MS), (LC/MS/MS)

The LC/MS portion of the platform was based on a Waters ACQUITY UPLC and a Thermo-Finnigan LTQ-FT mass spectrometer, which had a linear ion-trap (LIT) front end and a Fourier transform ion cyclotron resonance (FT-ICR) mass spectrometer backend. Fragmentation spectra (MS/MS) were typically generated in data dependent manner, but if necessary, targeted MS/MS could be employed, such as in the case of lower level signals.

#### Compound identification

Compounds were identified by comparison to library entries of purified standards or recurrent unknown entities. Identification of known chemical entities was based on comparison to metabolomic library entries of purified standards. The combination of chromatographic properties and mass spectra gave an indication of a match to the specific compound or an isobaric entity.

#### Statistical calculation

For pair-wise comparisons, we typically performed Welch’s t-tests. For other statistical designs t-test was performed. For creating PCA plots, the Clustvis free tool was used (https://biit.cs.ut.ee/clustvis/) [27]**.** For generating box plots, the freely available BoxPlot R (http://shiny.chemgrid.org/boxplotr/) was used. Except were indicated, at least three replicate experiments were performed to ensure significance. For correlation studies, we used an online tool https://mathcracker.com/correlation-coefficient-calculator or GraphPad (PRISM) (GraphPad Software, San Diego, CA 92108).

#### AA determination in cell media

The arachidonic acid ELISA Kit (UNES00032) (American Research Products Inc., Waltham, MA, USA) was used to semi-quantitate the amount of AA after the various treatments, according to the manufacturer’s instructions. Briefly, a microtiter well plate pre-coated with an AA was used and sample or standards were added to the wells along with a fixed quantity of biotinylated Arachidonic acid (competing with the endogenous one in the sample) and incubated. The absorbance at 450 nm was inversely proportional to the amount of Arachidonic acid, which was contained in the sample or standard.

#### Spheroid formation

For generating cell spheroids, variable number of single cells /well were seeded into BIOFLOAT™ 96-well plates (FaCellitate, Mannheim, Germany) in DMEM-F12/1:1 + Glutamax supplemented with BSA, bFGF (20 ng/ml) and hEGF (10 ng/ml) (Life Technologies, Grand Island, NY, USA). 

#### Spheroid diameter measurement

Briefly, spheroids grown in BIOFLOAT™ 96-well plates were spun on coverlisp slides with a Thermo Shandon Cytospin 3 Centrifuge (Marshall Scientific, Hampton, NH, USA) and fixed with 4% PFA for 5 min before imaging. Bright field images of at least 30 spheroids/experimental point were randomly acquired and longer diameter measured by IMAGE J (https://imagej.nih.gov/ij/index.html).

#### ALDH detection

ALDH activity was assessed by flow cytometry in MPM cell line subsets using ALDEFLUOR kit (Stem Cell Technologies Vancouver, BC, Canada) in accordance with the manufacturer’s instructions. Briefly, red blood cells were lysed and remaining cells were washed with PBS and Aldefluor assay buffer. The cells were incubated with BODIPY aminoacetaldehyde, which is converted into a fluorescent molecule (BODIPY aminoacetate) in the cytoplasm. Specificity of the fluorescence was shown using the specific ALDH inhibitor diethylaminobenzaldehyde (DEAB). To eliminate dead cells, cells were stained with viability stain Sytox-Red (Life Technologies Inc., Grand Island, NY, USA). Cell populations were identified using a using a Cytoflex flow cytometer (Beckman Coulter Life Sciences, Indianapolis, USA). Distinct Aldefluor-positive and Aldefluor-negative populations were revealed after excluding debris and dead cells.

#### Detection of IkBα in cell lysates by ELISA

For detecting of IkBα levels, an ELISA based assay (Human Total IkB-alpha DuoSet IC ELISA, DYC4299–2, R&D Systems, Inc. Minneapolis, MN, USA) was used, according to the manufacturer’s instructions.

#### Detection of DNA bound p65 by ELISA

For detecting of NFkB p65 levels, an ELISA based assay (NFkB p65 Transcription Factor Assay Kit -ab133112, ABCAM, Cambridge, UK) was used, according to the manufacturer’s instructions. For extract preparation, a Nuclear Extraction Kit, ab113474, ABCAM, Cambridge, UK) was used, according to the manufacturer’s instructions.

#### Detection of cPLA2 activity

For assessing the cPLA2 activity, we used the Cytosolic Phospholipase A2 Assay Kit (ab133090) (ABCAM, Cambridge, UK). To improve the specificity of measuring the cPLA2 (and not the sPLA2), SDS free lysates were prepared by sonication, according to the manufacturer’s instructions and the lysate was filtered through a molecular weight cut-off filter of 30,000 Da (Amicon UFC53030, Sigma-Aldrich, St. Louis, MO, USA). The measurements were performed by reading the absorbance (415 nm) at 15 min after addition of the cell lysate.

#### RNA extraction and quantitative PCR

Total RNA was extracted using the Trizol Reagent (Life Technologies I-20900 Monza, Italy). The first-strand cDNA was synthesized according to the manufacturer’s instructions (M-MLV RT kit, Invitrogen). Gene expression was measured by real-time PCR using the SYBR-Green assay (Cell Signaling Technology, Inc., Danvers, MA, USA ) on a 7900HT instrument (Thermo Fisher Scientific, MA, USA). Beta-actin was used as reference control. All the primers (Human qPCR Primer Pair kit) were commercially available from OriGene Technologies, Inc., Rockville, MD, USA).

## Results

### Mesothelial cells and mesothelioma cell lines are metabolically heterogeneous entities

**Fig. 1:**
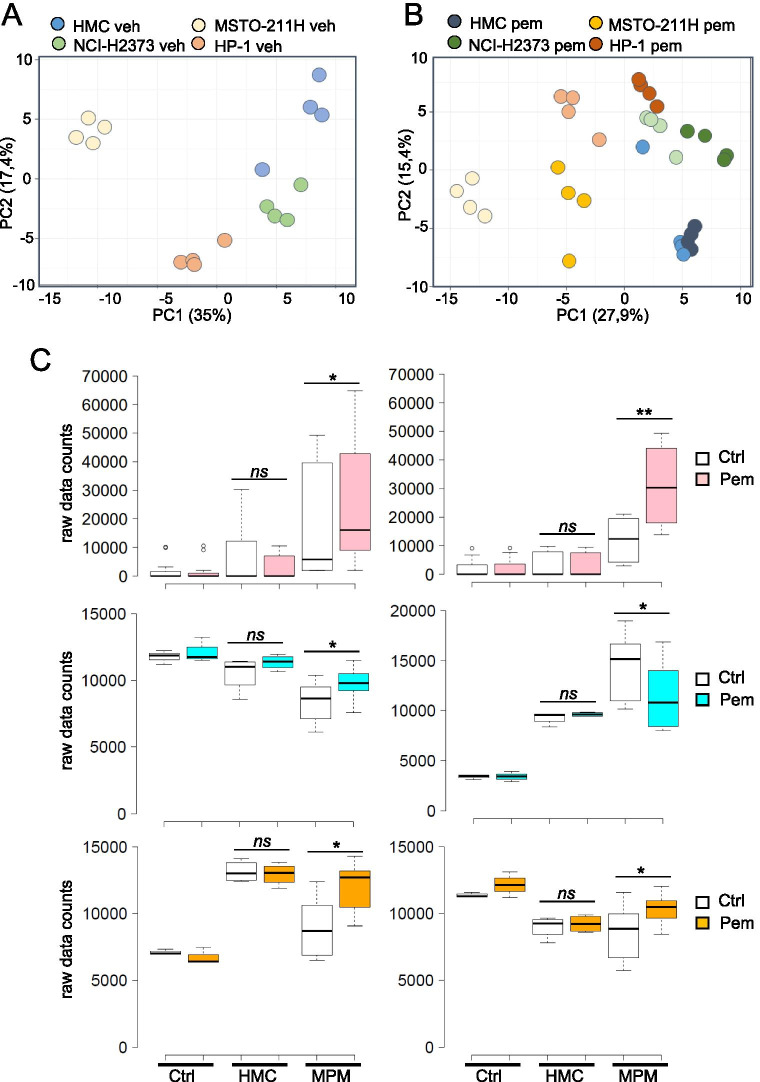
The transformation status affected the metabolic profile of mesothelial cells at steady state and after pemetrexed (pem) treatment. Mass spectrometry-based analysis of extracellular metabolites**. A.** PCA plot illustrating the distribution of human mesothelial untransformed cells (HMC, from 4 healthy donors) and of three mesothelioma (MPM) cell lines after ctrl (saline)-treatment for 24 h **B**. PCA plot showing the distribution of the same cell cultures after a short pulse of both ctrl and pemetrexed (pem) at a clinically relevant dose (see text, please). **C**. Representative box plots of three classes of metabolites selected because modulated in 3/3 MPM cell lines and unchanged in the HMC cultures after pem treatment. Upper panel: thymine (left) and uracil (right). Middle panel: glucose (left) and lactate (right). Lower panel: glutamine (left) and leucine (right). Please note that “ctrl” refers to spent media at 24 h in absence of cells (see methods please). Statistics: * *p* < 0.05. *ns* = not significant (*p* > 0.05)

We undertook a mass spectrometry approach to characterize metabolites differentially released in the extracellular medium by untransformed mesothelial (HMC) as opposed to mesothelioma (MPM) cells. Further, we focused on the ones increased or decreased upon pemetrexed (pem) treatment in ≥ two mesothelioma cell lines (*p* < 0.05). Conditioned media from ctrl (saline) - and pem-treated HMC and MPM cell lines (*n* = 3) were harvested at 24 h after a short pulse with ctrl or pem, at pharmacologically relevant concentration of the drug (42ugr/ml for 60 min, corresponding to the area-under-the-curve (AUC) of 500 mg/m^2^) [28]. To determine the background signal and the metabolite degradation occurring in absence of live cells, cell-free media samples collected at 24 h, in the presence or absence of pem, were included in the analysis. Principal Component Analysis (PCA) revealed a distinct clustering of ctrl-treated HMC samples and their transformed mesothelioma counterparts (Fig. 1A), suggesting that the transformation status was a determinant of the differences in metabolite distribution (Fig. 1A). Additionally, we observed a distinct separation between the ctrl– and pem-treated mesothelioma samples, with varying degree of effect (Fig. 1B). Such a separation was not observed for the untransformed HMC cells (Fig. 1B), indicating that the degree of metabolic perturbation between ctrl- and pem- treated mesothelial cells (HMC) was much lower than that between ctrl- and pem- treated mesothelioma cells. This matched the known therapeutic window of pem. We focused on the changes induced by the antifolate specifically in the chemoresistant mesothelioma cells with the intent of establishing a correlation between metabolite accumulation in the spent media and response to pem.

### Chemotherapy treatment affected the metabolite profile of mesothelioma cells

The analysis of the spent media from the pem treated mesothelioma cells revealed alterations in nucleotide metabolism, amino acid catabolism, representation of membrane lipids, and energy metabolism. We focused on those common to ≥ two mesothelioma cell lines and absent or not statistically significant in the untransformed mesothelial cells (HMC) (Fig. 1C).

#### Metabolic changes induced by pem treatment: nucleotide metabolism

Following pem treatment, NCI-H2373, MSTO-211H and HP1 cell media exhibited elevated levels of the purine nucleotides adenine and guanine. Similarly, the pyrimidine metabolites cytidine, thymine, and uridine were also elevated (Fig. 1C upper panels and Table S1). Higher levels of these metabolites (compared to control cultures without cells) may be indicative of a decreased utilization and increased release from the cell. We also observed accumulation of the downstream purine catabolic products xanthine and hypoxanthine as well as of the pyrimidine degradation products uracil and 2′-deoxyuridine in NCI-H2373, MSTO-211H, and HP1 media samples (Fig. 1C upper panels and Table S1). Thus, pem treatment induced an imbalance and subsequent release of nucleic acid metabolites inefficiently utilized by mesothelioma cells for nucleic acid synthesis. Such a finding echoes what observed in clinical studies with pem [5, 6].

#### Glucose metabolism

Compared to media alone, glucose levels were reduced in each of the samples (both mesothelial and mesothelioma ones) reflecting increased utilization by the cells (Fig. 1C middle panel left and Table S1). Pem treatment resulted in significantly higher levels of glucose in the media of MPM cells accompanied by significantly reduced levels of pyruvate (Table S1) and lactate (Fig. 1C, middle panel right). Together, these observations suggest that pem treatment inhibited glucose metabolism.

#### Amino acid metabolism

Methionine levels increased in each of the three-mesothelioma samples possibly resulting from reduced nucleic acid methylation (Table S1). Additionally, the branched chain amino acids (BCAA) isoleucine, leucine, and valine accumulated in media from pem- treated MSTO-211H and HP1 media (Fig. 1C lower panels and Table S1). Similarly, higher levels of the alpha-keto acids 4-methyl-2-oxopentanoate, 3-methyl-2-oxobutyrate, and 3-methyl-2-oxovalerate were observed in drug-treated NCI-H2373 and MSTO-211H media and this could be indicative of BCAA degradation as suggested by elevated propionyl-carnitine levels (Table S1). Other amino acids including glycine, aspartate, asparagine, glutamine, and glutamate were also higher in media isolated from NCI-H2373, MSTO-211H, and HP1 pem-treated cells respect to their ctrl-treated counterparts (Fig. 1C lower panels and Table S1). Altogether, these changes may reflect a reduced protein synthesis resulting from AMPK activation [7, 8]. Further, citrulline levels were elevated in media harvested from pem-treated NCI-H2373, MSTO-211H and HP1 cells (Table S1). These observations may be indicative of an increase in amino acid metabolism and activity of the urea cycle in these cultures, considering that citrulline was elevated compared to media alone. A reduction in the vitamin B6 related metabolites pyridoxal and pyridoxate (Table S1), cofactors in multiple amino acid reactions such as transamination and decarboxylation, also suggested a change in AA metabolism in pem-treated MPM cells.

#### Membrane lipids

Following pem treatment, we detected elevated levels of the phospholipid choline in MSTO-211H, and HP1 culture media (Table S1). Higher choline levels may be indicative of greater availability for phospholipid synthesis or enhanced phospholipid degradation. In support of phospholipid catabolism, glycerol-phosphorylcholine (GPC) and glycerol 3-phosphate (G3P) also were modestly elevated post-treatment (Table S1). Reduced glycerol levels in MSTO-211H and HP1 media indicated altered phospholipid hydrolysis (Table S1).

#### Lipid metabolism

**Fig. 2:**
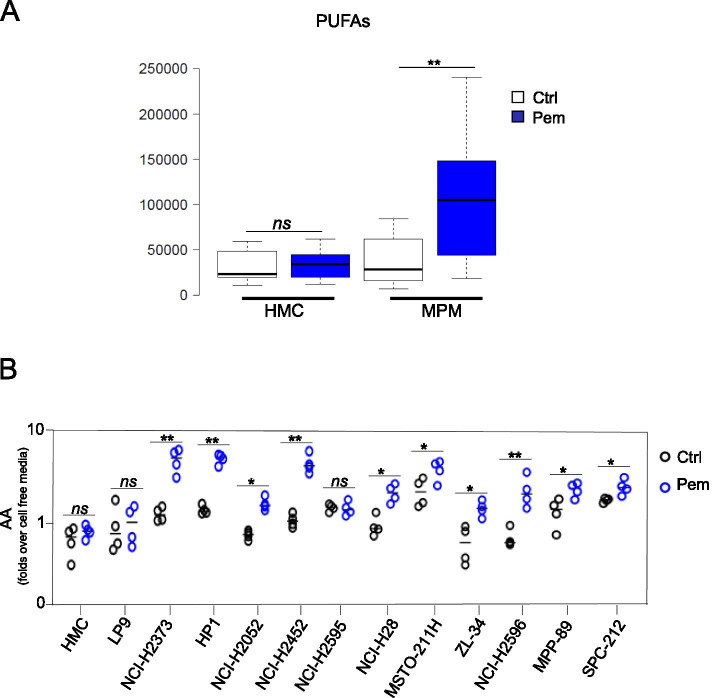
PUFAs were increased by pemetrexed treatment. **A**. Representative box plots of PUFAs (namely, arachidonate (20:4n6), eicosenoate (20:1n9 or 11) and docosahexaenoate (DHA; 22:6n3) as detected by mass spectrometry in the conditioned media from pem-treated cells. No changes in human untransformed mesothelial cells (HMC) cultures were recorded. **B**. ELISA-based detection of arachidonic acid (AA) in the conditioned media of the indicated MPM cell line treated with pemetrexed as indicated in Fig. 1A. AA levels were expressed as folds over cell-free media. No increase of AA in the pem-treated HMC or in the immortalized LP9 mesothelial cells was recorded. Statistics: * p < 0.05; ** *p* < 0.01. ns = not significant (p > 0.05)

Related to the latter observation, we recorded a statistically significant increase of polyunsaturated fatty acids (PUFAs) in the mesothelioma cell media (Fig. 2A), namely of arachidonate (20:4n6), docosahexaenoate (DHA; 22:6n3) and eicosenoate (20:1n9 or n11) (Fig. S1). Although differences in lipid availability may reflect altered oxidation, the ketone body 3-hydroxybutyrate (a marker of lipid oxidation) did not significantly differ between ctrl- and pem- treated cells (Table S1), suggesting that the differences in lipid accumulation could result from lipid hydrolysis in the pem-challenged MPM cells. We focused next on arachidonic acid (AA) because of its signaling potential in cancer cells [29] and because it was increased in 3 out of 3 MPM cell lines (while both eicoseanoate and DHA were not significantly increased in the MSTO-211H cell media, *p* > 0,05) (Fig. S1).

### Arachidonic acid accumulated in pem-treated mesothelioma cells but not in untransformed ones and this correlated with their sensitivity to pem

First, we validated the observed pem-instigated increase of AA by means of an unrelated technique (ELISA assay). This revealed that most of the cell line released basal levels of AA (as compared to cell free media) and ten out of eleven cell lines increased arachidonic acid release after a pem pulse (Fig. 2B). Neither the untransformed HMC cells nor the hTERT immortalized, untransformed mesothelial cells (LP9) [30] exhibited significantly increased AA levels after pem (Fig. 2B). This may indicate that AA release could be a feature of fully transformed cells.

### cPLA2 was responsible for freeing AA from membrane lipids after pem treatment

**Fig. 3:**
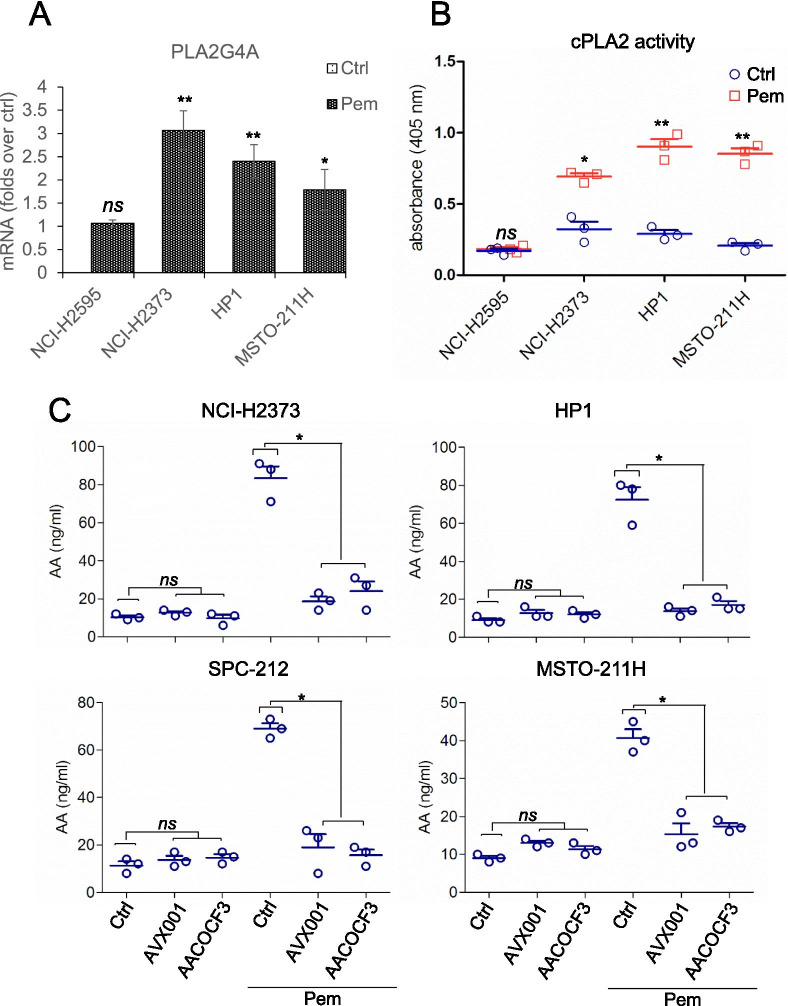
cPLA2 mediates the release of AA in pemetrexed-treated cells. **A**. Histogram showing the relative levels of PLA2G4A mRNA in ctrl- and pem-treated cells, at 24 h, as detected by RT-QPCR. **B**. cPLA2 activity detected by ELISA assay in the indicated cell lines treated with ctrl- or -pem for 24 h. C. Box plots showing the levels of AA in the indicated mesothelioma cell lines pretreated or not with two cPLA2 inhibitors for 90 min before being challenged with pem. Statistics: * *p* < 0.05; ** *p* < 0.01. *ns* = not significant (*p* > 0.05)

In this experimental setting, apoptotic events were undetectable at time of media harvesting (24 h) (Fig. S2), thus suggesting that AA was actively released rather than lost, because of the cell membrane damage following treatment. The group IVA phospholipase A2 (cPLA2α) is selective for arachidonic acid (AA) in the phospholipid sn-2 position, and is a major contributor to the increased levels of free AA in inflammation [31]. To evaluate the involvement of cPLA2 in the AA increase after pem treatment, we first assayed the levels of the cPLA2 (PLA2G4A) mRNA in four representative MPM cell lines (Fig. 3A). This revealed that all four cell lines expressed similar levels of the cPLA2 mRNA, which was unmodified by pem treatment at 24 h (Fig. 3A). Next, we tested, by ELISA, the enzymatic activity of the cPLA2 in the indicated cell lines. This showed a significant increase of the enzymatic activity after pem treatment (24 h), and no significant increase in the NCI-H2595 (Fig. 3B). This matched the absence of AA increase in the pem-stimulated NCI-H2595 cells (Fig. 2B). To further investigate the mechanism behind the AA increase in the media of pem-treated MPM cells, we evaluated the effect of inhibiting cPLA2 (Fig. 3C). To this aim, we pretreated the four representative MPM cell lines (NCI-H2373, HP1, SPC-212, MSTO-211H) with arachidonyl-trifluoromethyl ketone [32], or with AVX-001 [33], both specific phospholipase A2 (cPLA2) inhibitors (Fig. 3C), the latter one in clinical use for psoriasis [34]. We used both compounds at pharmacologically relevant concentrations (3micromol/L) [34, 35]. Pre-treatment (90 min) of MPM cells with both AACOCF3 and AVX-001 strongly attenuated the release of AA induced by pem treatment (Fig. 3C). Altogether, this suggested that cPLA2 could be responsible for freeing AA from membrane lipids after pem treatment.

### Arachidonic acid increased the ALDH^bright^ cell number

**Fig. 4:**
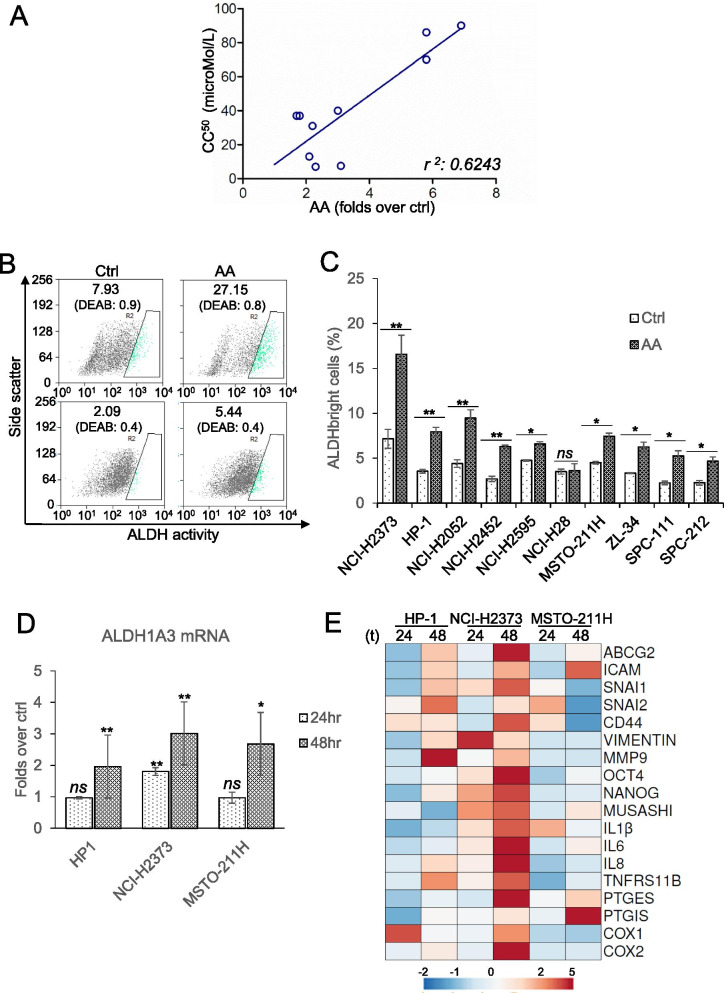
AA treatment increased the number of ALDH^bright^ MPM cells.** A.** Positive correlation between the CC50 of pemetrexed (y-axis) and the magnitude of AA release (expressed as folds over ctrl) in nine representative MPM cell lines (as from Fig. 2B, except that NCI-H2595 was not included because no significant increase in AA was recorded following pem treatment). Linear regression analysis with indicated r square (r^2^: 0.6243). **B**. Representative flow cytometry plots of NCI-H2373 (upper panels) and SPC-212 (lower panels) MPM cells treated for 48 h with AA 3 microMol/L or ctrl (saline), The percentage of ALDH^bright^ cells was determined over the same cells treated with a specific ALDH inhibitor (DEAB) immediately after adding the ALDH substrate (BAAA). **C**. Histogram showing the percentage of ALDH^bright^ cells treated as indicated in A, from additional representative MPM cell lines. Duplicate experiments. **D**. Histogram showing the relative levels of the ALDH1A3 mRNA detected by RT-QPCR in HP1, NCI-H2373, MSTO-211H cells treated with AA (3micromol/L), for 24 h. Folds over control-treated cells were reported. Statistics: * *p* < 0.05 except where indicated *ns* = not significant (*p* > 0.05). **E**. Heatmap showing the levels of the indicated mRNAs detected by RT-QPCR in HP1, NCI-H2373, MSTO-211H cells treated with AA (3microMol/L), for 24 and 48 h. Only mRNA significantly modulated (p < 0.05) at either 24 h or 48 h or both were included in the map

When searching for cell features correlated to AA release, a positive correlation between the amount of AA released and the experimentally validated CC_50_ of the donor MPM cell lines could be established (r^2^ = 0.6243; *p* = 0.015) (Fig. 4A). This raised the possibility that the released AA could be part of an adaptive cell response to the drug and prompted us to investigate whether treatment of MPM cells with pharmacologically relevant doses of AA would elicit cell resistance phenomena. First, we evaluated whether exogenous AA treatment could increase the percentage of ALDH^bright^ cells, a chemoresistant cells subpopulation that others and we have shown to survive chemotherapy (e.g. pem) in MPM and other solid tumors [36–38]. AA treatment at pharmacologically relevant doses, (3microMol/L) [39]) elicited a significant increase of ALDH^bright^ cells in 9 out of 10 representative MPM cell lines, as revealed by the FACS- based detection of ALDH activity after 24 h of AA treatment (Fig. 4B, C). Next, we evaluated whether AA treatment affected the expression of the ALDH1A3 isoform, shown by others and us as enriched in MPM samples and mediating the enzymatic activity detected by the FACS-based assay. QRT-PCR performed on HP1, NCI-H2373 and MSTO-211H cells revealed increased mRNA levels of ALDH1A3 after AA addition (Fig. 4D).

### AA increased the expression of CSC- and EMT-related genes

Rearrangement of ALDH^bright^ cells in chemotherapy-treated MPM cultures correlated with perturbed expression levels of cancer-stem-cell (CSC) - and epithelial-to-mesenchymal transition (EMT)-related markers [13–16], and with anchorage independent growth [16]. Therefore, we performed a limited QRT-PCR based screening for a subset of CSC- and EMT-related mRNA in three MPM cell lines treated at 24 h and 48 h, with ctrl or AA, respectively (Fig. 4E). Firstly, we found a time-dependent increase of mRNA levels of cytokines previously shown to arise in chemotherapy treated MPM cultures, namely IL1β, IL-6, IL-8 [13, 14] (Fig. 4E). Secondly, we found higher mRNA levels of EMT (vimentin, MMP9, CD44, ICAM1, SNAI1, SNAI2, TNFRS11B)- and CSC (ABCG2, OCT4, Nanog, Musashi)-related mRNAs (Fig. 4E), all shown to contribute a clonogenic, chemo-resistant phenotype in MPM and other solid tumors [40, 41]. Finally, we found increased mRNA of enzymes involved in downstream processing of AA, namely PGES, PTGIS, COX1 and COX2 [42](Fig. 4E). Despite variability was observed in the magnitude of effect and in the kinetic of modulation, those changes were common to all three MPM cell lines treated with AA (Fig. 4E). Altogether, this indicated that exogenous administration of AA fully mimicked the cell subpopulation rearrangement and the gene expression changes that others and we have described in chemotherapy-treated MPM cell cultures [13, 43–47].

### AA activated the NFKB pathway

**Fig. 5:**
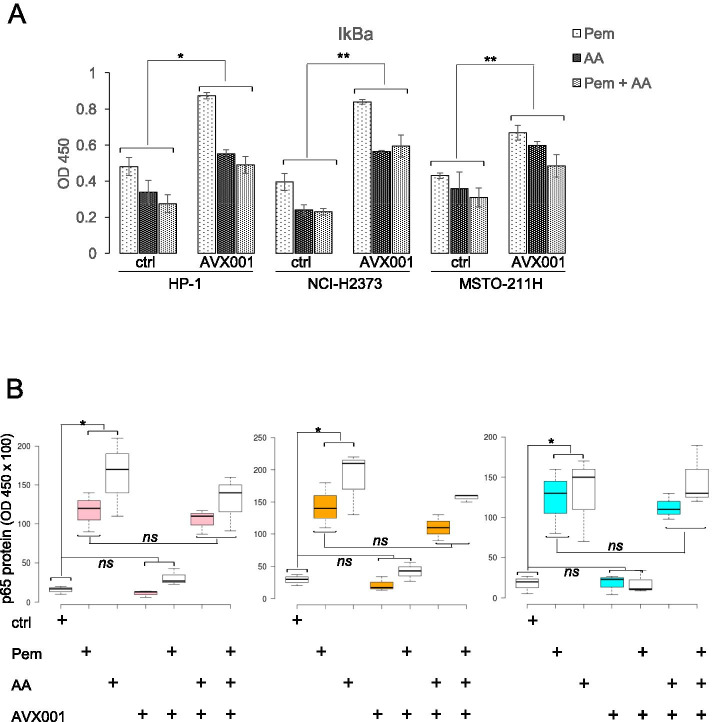
Pemetrexed-instigated release of AA activated NFkB.** A.** Protein levels of IKB detected by ELISA in MPM cells, pre-treated or not with a cPLA2 inhibitor (AVX001, 3 micromol/L for 90 min), and treated as indicated. Statistics: * *p* < 0.05 ** *p* < 0.01. *ns* = not significant (*p* > 0.05). **B**. Representative box plots showing the nuclear content of the NFkB subunit p65 bound to a synthetic NFkB responsive element and detected by ELISA in cell extracts of HP1 (left panel), NCI-H2373 (middle panel) and MSTO-211H (right panel). Statistics: * *p* < 0.05; *ns* = not significant (*p* > 0.05)

The expression of ALDH1A3 and the consequent increase of the ALDH^bright^ cell number in pem-treated MPM cells are mediated at least in part by NFkB activation [16, 48] and many of the genes shown here as upregulated by AA are known (IL-1β, IL-6, IL-8, SNAI1, ICAM1, COX-2, PTGES, PTGIS) [49–55] or putative (CD44, VIM, MMP9) (https://bioinfo.lifl.fr/NF-KB/) NFkB targets. Additionally, in non-small-cell lung cancer (NSCLC), pem was shown to activate the NF-κB signaling pathway, via a ROS- mediated IkBα degradation [56] and it was also shown to increase NFkB-p65 phosphorylation (Ser468) in MSTO-211H cells [57]. Thus, we investigated whether AA treatment for 24 h could modulate the NFkB pathway in MPM cells. We treated three MPM cell lines (HP1, NCI-H2373 and MSTO-211H) with pem and assessed, by means of ELISA assays, the NFkB activation (Fig. 5A, B). First, we found decreased levels of the inhibitor of NFkB alpha (IkBα) [58] in response to both pem and AA treatment, alone or combined, at 24 h (Fig. 5A). The effect of pem was readily attenuated by pre-treating the cells with AVX001 (90 min at 3microMol/L), while the IkBα downregulation in response to AA or pem + AA after cPLA2 inhibition was unchanged (Fig. 5A). Next, we found that pem increased the fraction of the p65 subunit of NFkB bound to a synthetic oligonucleotide containing the NFkB DNA binding site, in MPM nuclear lysates (Fig. 5B). The cPLA2 inhibitor AVX001 strongly attenuated the increase of p65 (Fig. 5B), while exogenous AA addition (10microMol/L) promptly rescued the AVX001 affect (Fig. 5B). On the other hand, AA increased the p65 accumulation in an AVX001-insensitive manner. Further, adding pem to AA only slightly increased the effect of AA alone (Fig. 5B).

### AA and not its COX- and LOX-derivatives mediated the early effect on MPM cells

**Fig. 6:**
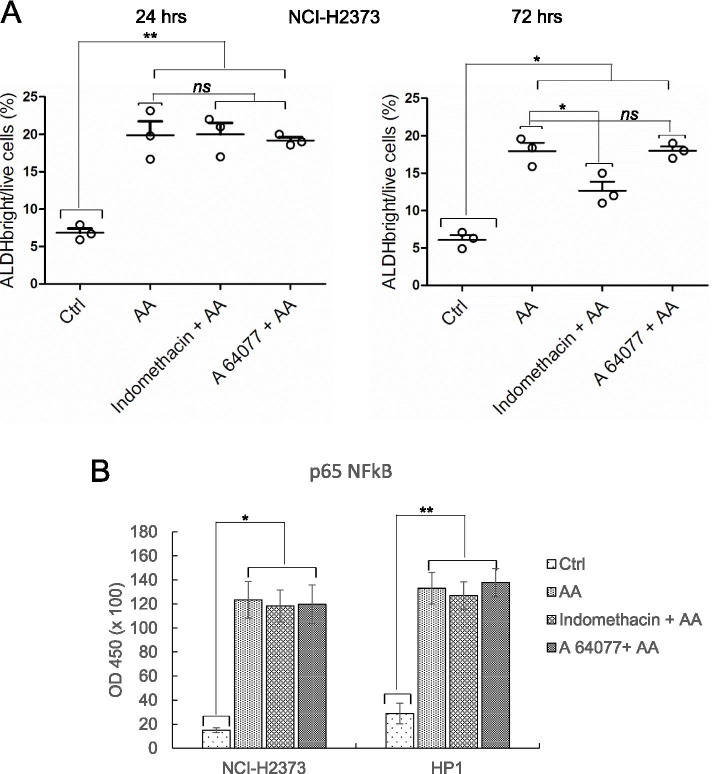
Arachidonic acid and not its downstream metabolites, mediated the early increase of ALDH^bright^ cells and the NFkB activation. **A**. Representative graphs showing the percentage of NCI-H2373 ALDH^bright^ cells detected by FACS analysis after addition of ctrl or AA, in absence or presence of pretreatment with indomethacin (10 micromol/L) and A-64077 (Zileuton) (5 micromol/L), 90 min before addition of AA. Left panel; percentage of ALDH^bright^ cells detected 24 h after addition of the indicated drugs. Right panel: percentage of ALDH^bright^ cells detected 72 h after addition of the indicated drugs. **B**. Representative graphs showing the levels of p65 NFkB detected by ELISA in the NCI-H2373 and HP1 MPM cells treated for 24 h as indicated in 6A. Statistics: * p < 0.05 ** p < 0.01 ns = not significant (p > 0.05)

AA is known to be quickly metabolized, after its release, by cyclooxygenases (COX) and Lipoxygenases (LOX), to generate bioactive molecules [59]. This raises the possibility that the COX- and LOX-mediated downstream processing of AA could mediate the effect of AA. To understand this, we evaluated the involvement of COX and LOX in two main experimental settings. First, we evaluated the effect of indomethacin (NSC-77541), a COX1/2 inhibitor and of A-64077 (Zileuton), a 5-LOX inhibitor [60], to affect the AA-induced increase of the NCI-H2373 ALDH^bright^ cells (Fig. 6A). FACS–based count of ALDH^bright^ cells in time revealed that pretreatment with indomethacin (10 microMol/L) or A-64077 (5 microMol/L), did not affect the AA-mediated increase of ALDH^bright^ cells, after 24 h of treatment (Fig. 6A). When assaying the ALDH^bright^ cells at later time points (72 h), we found a moderate effect of COX inhibition on the percentage of ALDH^bright^ cells (Fig. 6A, right panel, *p <* 0.05). We observed similar results when identically treating the HP1 cells (Fig. S3). This may suggest that, at later time points, COX-mediated processing of AA does contribute to the increase of ALDH^bright^ cells. Further, down on this line, we investigated whether COX and LOX inhibitors could attenuate early NFkB activation. ELISA assays revealed that at 24 h, no differences in the levels of the oligonucleotide trapped-p65 could be observed among the samples treated with AA or pretreated with indomethacin or A-64077 before AA addition, in both NCI-H2373 and HP1 cells (Fig. 6B). Altogether, these observation suggest that unprocessed AA, released by c-PLA2, is responsible or the early effect on the MPM cells. This matched the absence of PGE2 detected in the spent media from the pemetrexed –treated cells lines (Table S1).

### AA modulated 3D spheroid formation

**Fig. 7:**
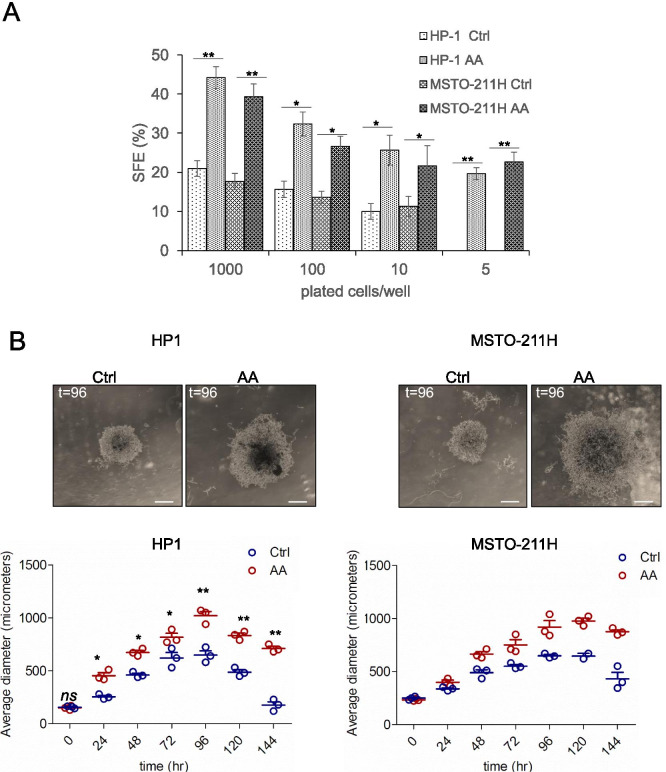
Arachidonic acid modulated the sphere forming ability of MPM cells. Briefly, MSTO-211H and HP1 cells were clonally seeded in presence or absence of AA (3micromol/L) and allowed to grow for 7 days. **A**. Graphs showing quantitation of the sphere forming efficiency (SFE) from MSTO-211H (left panel) and HP1 (right panel) cells treated with ctrl or AA, at different seeding densities. Statistics: * *p* < 0.05 ** *p* < 0.01 *ns* = not significant (*p* > 0.05). **B**. Upper panel: representative micrographs of the formed spheroids (HP1, left; MSTO-211H, right) at day 4 from ctrl - and AA -treated cultures. Scale bar: 200 μm. Lower panel: graphs reporting the average (longer) diameter of the HP1 and MSTO-211H-derived spheroids in time. Statistics: * p < 0.05 ** p < 0.01 ns = not significant (p > 0.05)

Propagation in 3D conditions and in absence of adherence is a pro-tumorigenic feature and the ability to do so correlated with the enrichment for CSC and EMT-related genes and increased resistance to drug treatment [14, 61, 62]. Additionally, we have shown that conditioned media from chemotherapy-treated MPM cultures favored spheroid formation in recipient untreated cultures [14]. Thus, we investigated whether AA treatment could instigate or modulate 3D spheroid formation of mesothelioma cells. Treatment with AA for 6 days after single cell plating in absence of serum triggered a significant increase of the number of MSTO-211H- and HP1- derived spheroids (Fig. 7A). In detail, calculation of the sphere forming efficiency (SFE) revealed a significant increase in the sphere-forming-ability (SFE) of the AA-treated cells as compared to the ctrl- treated ones (Fig. 7A). At the lowest cell seeding density, we recorded spheroid formation only in AA-treated cultures (Fig. 7A). Additionally, measuring the average longer diameter of the formed spheroids revealed a significant effect of AA on the size of those structures (Fig. 7B, upper left and right panels, respectively),, which persisted in time (Fig. 7B, lower left and right panels, respectively).

#### cPLA2 inhibition impaired 3D spheroid formation and sensitized sphere-forming-cells to pem

**Fig. 8:**
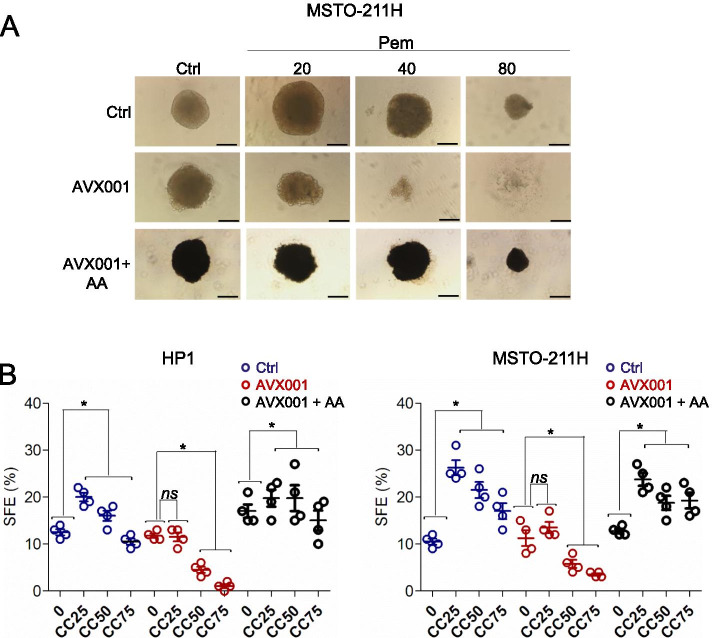
cPLA2 inhibition blunted the effect of pemetrexed on spheroid formation. Briefly, MSTO-211H cells were clonally seeded in spheroid-forming conditions, in presence or absence of the cPLA2 inhibitor (AVX001 3micromol/L) (upper and middle panels) or in presence of AVX001 + AA (lower panels) and allowed to grow for 7 days, after treatment with ctrl or pem at increasing doses (20–80 micrograms/ml/1 h), before washing out of the drugs. **A**. Representative micrographs of the formed spheroids at day 5. Scale bar: 200 μm. **B**. Graphs showing quantitation of the Sphere Forming Efficiency (SFE) from HP1 (left) and MSTO-211H (right) cells seeded and treated as in A (1000 cells/well seeded). Please note that little to no spheroid formation was observed at higher doses of pem, in presence of the cPLA2 inhibitor and that adding back AA rescued cPLA2 inhibition

Next, we set out to evaluate whether inhibition of cPLA2, hence of AA release, would attenuate the resistance of the sphere forming cells to pem. As shown already by others and us [13, 16, 63–65], HP1- and MSTO-211H-derived spheroids were resistant to pem over a broad range of doses (20–80 micrograms/ml/1 h) (Fig. 8A). Pre-treatment with AVX001 strongly affected size (Fig. 8A) and number (Fig. 8B), of the spheroids, mostly at higher pem doses. In detail, evaluating the SFE after seeding 1000 cells/well revealed a profound effect of cPLA2 inhibition on the SFE, which was readily reversed by exogenously added AA (10micromol/L), for both HP1 (Fig. 8B, left panel) and MSTO-211H (Fig. 8B, right panel)-formed spheroids. Altogether, this indicated that a pem-instigated cPLA2-AA-NFkB axis might instigate key pro-tumorigenic features of MPM cells.

## Discussion

Others and we have demonstrated that a complex mix of cytokines and growth factors sustains the emergence of cancer stem cell-like cell subpopulations in chemotherapy- treated cell lines and primary cultures, according to a Senescence-Associated-Secretory-phenotype (SASP) model [11, 13, 15]. However, onset of SASP takes days to weeks to occur [13, 14, 66, 67] therefore we searched for earlier mediators of such a process. We tested here the hypothesis that metabolic remodeling in pemetrexed (pem) -treated cells could initiate the SASP-driven secretome rearrangement. Within such an experimental frame, we firstly focused on the metabolites differentially released by pem-treated mesothelial and mesothelioma cells. This revealed a significant impact of the transformation status on the extracellular metabolome of the cells, as revealed by the PCA analysis, at steady state (Fig. 1A). With regard to the pem treatment, we have used drug treatment conditions as much as possible closed to clinical relevance, compatible with the therapeutic window of the compound in clinical settings [6, 28]. As a result, we found no effect of pem treatment on the viability of untrasformed, mesothelial cells (HMC). On the other hand, we showed effective target engagement in the mesothelioma cell lines, in that pem treatment increased the amount of unused purine and pyrimidine, consequent to the impaired DNA and RNA synthesis (subsequent to the inhibition of thymidylate synthase, dihydrofolate reductase and glycinamide ribonucleotide formyl-transferase [6]. We also observed reduced utilization of aminoacids, and decreased usage of glucose and lactate. All these changes may be compatible with the activator effect of pem on AMPK [7, 8]. Altogether, this confirmed specificity of action of the drug, matching its known or expected actions. We found a significant increase of PUFAs in pem–treated mesothelioma cells (and not in the mesothelial ones) and we focused on the long chain fatty acid arachidonic acid for its being broadly increased in 9/10 MPM cell lines after pem treatment and its emerging role in cancer signaling [68, 69]. We found that a cPLA2-mediated increase of AA after pem induced features of NFkB activation, modulated the number of ALDH^bright^ cells, the expression of CSC- and EMT-related transcripts and the 3D growth of the pem-treated MPM cells. In summary, early release of AA executed most of the pro-tumorigenic actions others and we have described in pem- and chemotherapy-treated cancer cells. Further, AA modulated, in MPM cells, the levels of IL1β, IL-6 and IL-8, main contributors to the SASP-driven chemoresistant phenotype [11, 13, 16]. This enforces the possibility that cPLA2-mediated AA release by pemetrexed and its effect on NFkB activation represent early events of the SASP-driven secretome rearrangements. This rearrangement of cell subpopulations correlated with chemo resistance in MPM and other solid tumors [13, 70] and, in facts, here we demonstrated that cPLA2 inhibition sensitized the spheroid forming cells to the effect of pemetrexed (Fig. 6).

There is growing evidence that AA may mediate adaptive stress responses. For example, in colorectal cancer, AA treatment promoted expansion of radioresistant Musashi^pos^ cells in irradiated mice [24], suggesting involvement of AA in the adaption to therapy-induced stress. Since the MPM ALDH^bright^ cells are known to be radio- [71], chemo-resistant [13, 44] and Musashi^pos^ [72, 73], we speculate that in CRC, AA elicited a redistribution of cell subpopulations remarkably close to what observed in our experimental system. This is also in line with the fact that attenuating lipid desaturationimpaired cancer stem cell-associated pathways, like in ovarian cancer (OC) spheroids [20]. SCD1 inhibition reduced expression of ALDH1A1, Nanog, and Oct4 in NSCLC [74] and reverted chemo-resistance of BRAF^pos^ melanoma cells [75].

We have not observed, in the adopted experimental settings, significant apoptosis or cell damage (up to 24 h-time of media harvesting) (Fig. S2), arguing against a passive release of AA due to cell damage. Instead, we found that cPLA2 was mostly responsible for releasing AA in pem-treated cells. An open question here is what activated cPLA2 after pem treatment. Recently, Dr. Lu and coworkers have shown that a ROS-dependent activation of cPLA2 takes place consequent to inhibition of thymidylate synthase (TS), in pem-treated NSCLC [56]. The metabolomic changes we have observed here are compatible with TS inhibition (Table S1). This suggest that a similar modality of cPLA2 activation may take place in MPM. Worth mentioning, there is also the possibility that AA stimulated cPLA2 activity in a feed-forward manner: for example, arachidonic acid induced cPLA2 in prostate carcinoma cells [76]. We have not tested this possibility here.

The early changes observed in the protumorigenic traits of MPM cells after AA addition are likely to be mediated by unmodified AA, since COX and LOX inhibition did not reduce the effect of AA at 24 h of treatment. Further, the spent media, harvested at 24 h did not contain detectable AA-derivatives, such as PGE2 (Table S1). On the other hand, we observed an effect of COX inhibition on the AA action at 72 h (Fig. 6A), thus suggesting that COX-mediated processing of AA may contribute to the modulation of chemoresistance. This matches the increased COX1 and COX2 mRNAs after AA treatment (Fig. 4E) and the prognostic value of COX2 protein expression in MPM [77].

Expression of cPLA2 correlated with metastatic behavior and poor prognosis in osteosarcoma by facilitating EMT [78] and evidence is growing regarding pro-tumorigenic functions of cPLA2 [79]. Further, on this note, PI3KC mutant-carrying breast cancer cells released AA in a cPLA2-dependent way, thus suggesting that cPLA2 activity may be a target of cancer promoting events [80]. Although we did not observe stratification value of PLA2G4A mRNA levels when interrogating the MPM TGCA repository (data not shown), we have not investigated here the protein levels or its post-translational status in MPM specimens. This certainly prompts for future studies.

Finally yet importantly, the data shown here may be relevant for other chronic inflammation-related neoplasms. For example, the levels of AA in peritoneal fluids from ovarian carcinoma patients were shown to hold prognostic value [81] and a recent “omics” profiling of ovarian cancer-derived, AA treatment of mononuclear cells revealed important pathway activation, among all deregulation of MAPK and Rho-GTPase signaling [82]. Additionally AA secreted by adipocytes, inhibited cisplatin-induced apoptosis of ovarian cancer cells [83], further supporting our observations. This suggests a bidirectional signaling between cancer cells and the tumor microenvironment, which may be tumor- stage and -state specific.

## Conclusions

In this experimental work, we have shown that early, c-PLA2 dependent release of AA, from pemetrexed-treated MPM cells, activates NFkB and triggers gene expression and cell subpopulation rearrangements compatible with a chemoresistant phenotype. This establishes a missing link between therapy-instigated lipid signaling and the complex stress adaptive program leading to chemoresistance. Our findings may be of translational relevance because of the availability of clinical trial grade cPLA2 inhibitors whose ability to counteract MPM chemoresistance could be explored, in the next future, in combinatorial settings.

## Supplementary Information

**Figure S1. PUFAs were increased in MPM supernatant after pem treatment.** Histograms showing the levels of arachidonate (20:4n6) (upper panel), docosahexaenoate (DHA; 22:6n3) (middle panel), eicosenoate (20:1n9 or 11) (lower panel) in the ctrl- or pem-treated MPM cell lines indicated. Statistics: * p<0.05 ** p< 0.01. ns= not significant (p>0.05). (PPTX 62 kb)

**Figure S2. No significant apoptosis followed pem treatment at 24hrs.** Viability assay. Histograms showing the percentage of Sytox Blue negative cells at 0 and up to 96hrs after pem treatment. Statistics: * p<0.05. ns= not significant (p>0.05). (PPTX 632 kb)

**Figure S3. Arachidonic acid and not its downstream metabolites , mediates the early increase of ALDH**^**bright**^ **cells and the NFkB activation.** Representative graphs showing the percentage of HP1 ALDH^bright^ cells detected by FACS analysis after addition of ctrl (saline), AA, in absence or presence of pre-treatment with indomethacin (10 microMol/L) and A-64077 (Zileuton) (5 microMol/L) (90 minutes before addition of AA). Left panel; percentage of ALDH^bright^ cells detected 24 hrs after addition of the indicated drugs. Right panel: percentage of ALDH^bright^ cells detected 72 hrs after addition of the indicated drugs. Statistics: * p<0.05. ns= not significant (p>0.05). (PPTX 233 kb)

**Table S1..** Heat map of the biochemicals profiled in this study. Mean values expressed as fold of change among ctrl- and pemetrexed treated- samples after scaling and background subtraction were expressed with different colors, as statistically significant (green, red) or as approaching significance (pink, light green), respectively. The ratio between spent media and cell free media for ctrl- and pem-treated cells is reported as well to show metabolite variations independent from the presence of cultured cells.

## Data Availability

All data generated or analyzed during this study are included in this published article [and its supplementary information files].

## Abbreviations

ALDH: Aldehyde Dehydrogenase
MPM: Malignant Pleural Mesothelioma
ELISA: Enzyme-linked immunosorbent assay
NFkB: nuclear factor of kappa light polypeptide gene enhancer in B-cells
IkBalpha: NFkB inhibitor, alpha
cPLA2: Cytosolic Phospholipase A2
sPLA2: Secretory Phospholipase A2
CSC: Cancer Stem Cells
EMT: Epithelial to Mesenchymal Transition
AA: Arachidonic acid
PUFAs: Poly Unsaturated Fatty Acids
TME: Tumor Micro Environment
