# Supplementary material for: Arachidonic acid drives adaptive responses to chemotherapy-induced stress in malignant mesothelioma
Source: J Exp Clin Cancer Res. 2021 Nov 2;40:344. doi: 10.1186/s13046-021-02118-y (PMC8561918; doi:10.1186/s13046-021-02118-y)
Supplement: Supplementary file 1 — Figure S1. PUFAs were increased in MPM supernatant after pem treatment. Histograms showing the levels of arachidonate (20:4n6) (upper panel), docosahexaenoate (DHA; 22:6n3) (middle panel), eicosenoate (20:1n9 or 11) (lower panel) in the ctrl- or pem-treated MPM cell lines indicated. Statistics: * p<0.05 ** p< 0.01. ns= not significant (p>0.05). (PPTX 62 kb) [file 13046_2021_2118_MOESM1_ESM.pptx]

## Slide 1
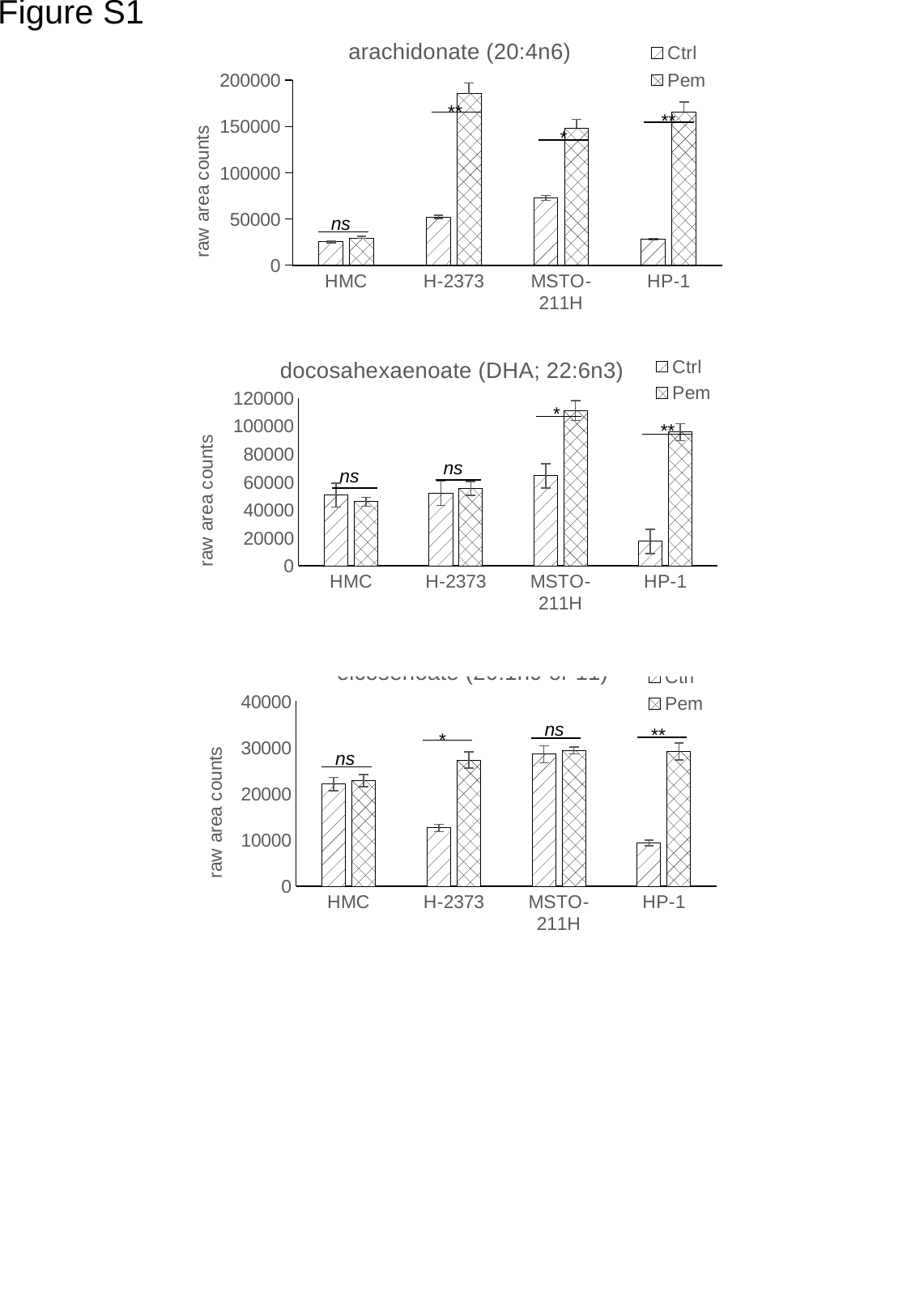

Figure S1
### Chart: arachidonate (20:4n6)
| Category | Ctrl | Pem |
|---|---|---|
| HMC | 25381.325759999996 | 29145.40346666667 |
| H-2373 | 52184.44107333333 | 185276.21906666667 |
| MSTO-211H | 72851.54902666667 | 148223.09276666667 |
| HP-1 | 28288.95178666667 | 165651.0022 |**
**
*
ns
### Chart: docosahexaenoate (DHA; 22:6n3)
| Category | Ctrl | Pem |
|---|---|---|
| HMC | 50618.63976666667 | 45853.115633333335 |
| H-2373 | 51948.54143333333 | 55459.98076666667 |
| MSTO-211H | 64441.611866666666 | 111080.3595 |
| HP-1 | 17590.079966666668 | 95681.13113333333 |*
**
ns
ns
### Chart: eicosenoate (20:1n9 or 11)
| Category | Ctrl | Pem |
|---|---|---|
| HMC | 22097.801033333337 | 22876.053233333336 |
| H-2373 | 12607.4678 | 27285.7087 |
| MSTO-211H | 28547.953233333334 | 29362.289066666664 |
| HP-1 | 9336.942633333332 | 29137.758166666667 |ns
**
 *
ns
