# Supplementary figures and images for: Arachidonic acid drives adaptive responses to chemotherapy-induced stress in malignant mesothelioma
Source: J Exp Clin Cancer Res. 2021 Nov 2;40:344. doi: 10.1186/s13046-021-02118-y (PMC8561918; doi:10.1186/s13046-021-02118-y)

## Slide 1
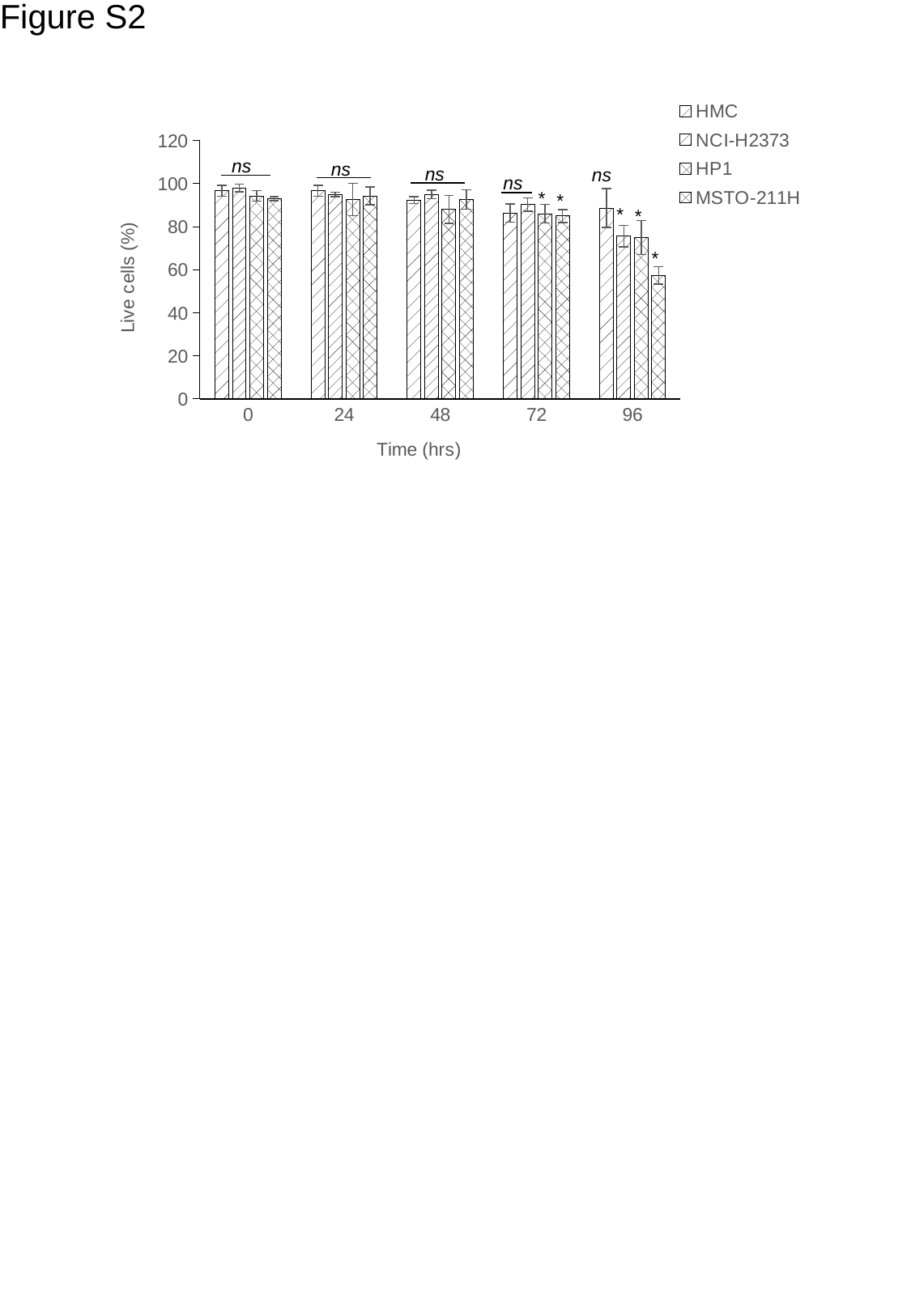

Figure S2
### Chart
| Category | HMC | NCI-H2373 | HP1 | MSTO-211H |
|---|---|---|---|---|
| 0 | 96.66666666666667 | 98.0 | 94.33333333333333 | 93.0 |
| 24 | 96.66666666666667 | 95.0 | 92.6666666666667 | 94.33333333333333 |
| 48 | 92.33333333333333 | 95.0 | 88.0 | 92.66666666666667 |
| 72 | 86.33333333333333 | 90.33333333333333 | 86.0 | 85.0 |
| 96 | 88.6666666666667 | 75.66666666666667 | 75.0 | 57.333333333333336 |ns
ns
ns
ns
ns
*
*
*
*
*

Supplement: Supplementary file 2 — Figure S2. No significant apoptosis followed pem treatment at 24hrs. Viability assay. Histograms showing the percentage of Sytox Blue negative cells at 0 and up to 96hrs after pem treatment. Statistics: * p<0.05. ns= not significant (p>0.05). (PPTX 632 kb) [file 13046_2021_2118_MOESM2_ESM.pptx]

## Slide 1
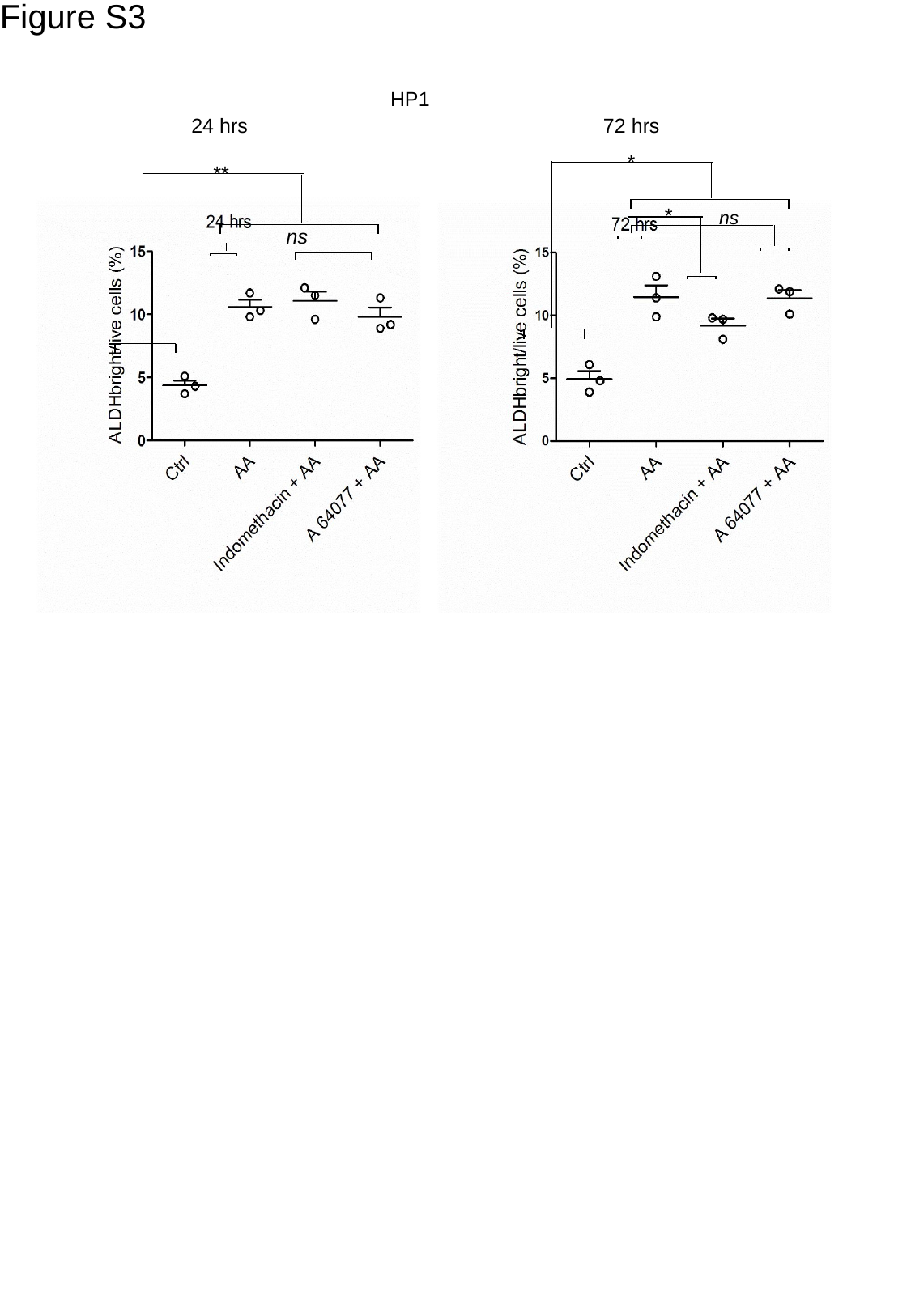

Figure S3
HP1
24 hrs
72 hrs
*
**
*
ns
ns

Supplement: Supplementary file 3 — Figure S3. Arachidonic acid and not its downstream metabolites , mediates the early increase of ALDHbright cells and the NFkB activation. Representative graphs showing the percentage of HP1 ALDHbright cells detected by FACS analysis after addition of ctrl (saline), AA, in absence or presence of pre-treatment with indomethacin (10 microMol/L) and A-64077 (Zileuton) (5 microMol/L) (90 minutes before addition of AA). Left panel; percentage of ALDHbright cells detected 24 hrs after addition of the indicated drugs. Right panel: percentage of ALDHbright cells detected 72 hrs after addition of the indicated drugs. Statistics: * p<0.05. ns= not significant (p>0.05). (PPTX 233 kb) [file 13046_2021_2118_MOESM3_ESM.pptx]
